# Exploring Biodegradable Polymeric Nanocomposite Films for Sustainable Food Packaging Application

**DOI:** 10.3390/polym17162207

**Published:** 2025-08-13

**Authors:** Nikolay Estiven Gomez Mesa, Alis Yovana Pataquiva-Mateus, Youhong Tang

**Affiliations:** 1 Department of Engineering, Universidad de Bogotá Jorge Tadeo Lozano, Bogotá 111711, Colombia; gome0105@flinders.edu.au; 2 College of Science and Engineering, Flinders University, Adelaide, SA 5042, Australia; 3 College of Medicine and Public Health, Flinders University, Adelaide, SA 5042, Australia

**Keywords:** bio-nanocomposite, casein, starch, bentonite, biopolymeric film, polyvinyl alcohol

## Abstract

In this study, a bio-nanocomposite integrating calcium caseinate, modified starch, and bentonite nanoclay was formulated and synthesized into film form via solution casting. Glycerol was incorporated for plasticization, and polyvinyl alcohol (PVA) was used to enhance the structural and chemical attributes of the material. The addition of PVA and bentonite notably improved the mechanical strength of the casein-based matrix, showing up to a 30% increase in tensile strength compared to similar biopolymer formulations. Water vapor permeability was significantly reduced when compared to previously reported casein–starch formulations, evidencing the barrier-positive effects of bentonite nanostructures. The microbial analysis confirmed that the quantity of bacterial colonies remained within permissible levels for non-antimicrobial biodegradable films; however, further antibacterial evaluations are advised. Biodegradability testing showed a consistent degradation trend, with full disintegration extrapolated to occur around 13 weeks under natural soil conditions. This study offers exploratory insight into the development of functional and biodegradable films using biopolymer blends and nanoclay suspensions, highlighting their potential in sustainable food packaging applications.

## 1. Introduction

As the search for biodegradable alternatives to plastics intensifies, the development of efficient packaging technologies is reshaping the future of food packaging [1]. The widespread use of plastic in everyday life, amplified by a growing global population, has led to an annual production exceeding 380 million tonnes [2], resulting in significant adverse environmental effects of plastic waste [3]. Researchers widely acknowledge that packaging polymers contribute substantial ecological risks due to their prolonged degradation times, which can often take several decades to centuries for complete breakdown [4]. Beyond the challenge of natural degradation, many developing countries lack advanced technological infrastructure and effective regulatory frameworks for the production, use, and management of plastic waste [5,6]. With up to 43% of this waste ending in landfills and limited knowledge surrounding disposal and soil remediation [7], the search for biodegradable and affordable materials has emerged as a key strategy for improving waste recovery and mitigating the associated environmental impact. Among these alternatives, bioplastics—obtained partially or totally from bio-based materials—have gained attention as a sustainable solution due to their natural degradation process [8]. Biodegradable polymers can be produced from biomass extraction, which includes neutral polysaccharides (e.g., cellulose), cationic polysaccharides (chitosan), anionic polysaccharides (alginate), polypeptides (gelatin), polyphenols (lignin) [9], lipids, and proteins (milk and soy proteins) [10]. Additionally, polyesters can be produced by microorganisms (polyhydroxyalkanoates) [11] and bio-based monomers synthesized from petroleum mixtures, such as copolyesters, e.g., poly (vinyl alcohol) (PVA) and polycaprolactone (PCL) [12].

Within this variety of bio-derived materials, proteins stand out for their versatility in diverse applications, offering moderate mechanical properties, excellent optical features, gas permeability, and fat barrier performance [13]. Their inherent structural and functional properties make natural proteins ideal for forming food-grade films [14]. With the ability to form dense molecular networks and exhibit strong covalent interactions, protein biopolymers have demonstrated effectiveness in preserving the freshness of minimally processed fruits and vegetables [15]. Thereby, naturally derived substances—such as casein and starch—have been investigated for their non-toxic nature and potential to produce biodegradable films [16,17].

Approximately 80% of the protein content in bovine milk consists of casein subunits, with the remaining 20% comprising various whey proteins [18]. Casein forms self-assembled colloidal structures known as micelles—spherical aggregates ranging from 50 to 400 nm in diameter—primarily composed of αS1-, αS2-, β-, and κ-casein [19,20]. Through its ability to bind ions and small molecules, casein enables the formation of caseinates, including calcium and sodium salts, which are commonly extracted from dairy industry products [21]. In addition, its high molecular flexibility supports structural integrity [22], while instability near its isoelectric point leads to gel formation during acidification [23]. Due to its non-toxic nature, high availability, and thermal stability [19,24], casein is a versatile material for developing edible films and packaging coatings [25]. Moreover, its random coil structure allows film formation, promoting strong intermolecular interactions, including electrostatic, hydrophobic, and hydrogen bonding [26].

Casein-based films have demonstrated diverse advantages across industries. In the biomedical field, they have been investigated for wound dressings [27] and pharmaceutical tablet coatings [28]. In the food sector, applications include fruit surface treatments [29], cheddar cheese packaging [30], and advanced food packaging formulations designed for the controlled delivery and transport of nutraceuticals [31]. The abundance of multiple polar functional groups (amino and hydroxyls) enables casein to impart effective barrier properties to the polymer film matrix against oxygen and non-polar molecules [32]. Additional features of food packaging include antioxidant and antihypertensive properties under various treatments [33], high water-binding capacity [34], and the ability to incorporate active ingredients to enhance functional properties [25]. Nevertheless, the drying process often causes contraction in pure casein films, becoming brittle after drying [32]. This limitation can be addressed by adding edible plasticizers, such as glycerol, which enhance film flexibility by increasing the free volume within the polymer matrix [32]. Therefore, further improvements can be achieved by blending nature-based polymers with agents and other biodegradable polymers [10], resulting in materials with distinct attributes.

Similarly, starch-based films have been recognized as eco-friendly alternatives to synthetic polymers, exhibiting biodegradability and high viability [17], making them promising candidates for cost-effective bio-composite packaging materials. Starch is anticipated to emerge as one of the most prominent biological polymers due to its abundance and significance in bio-based materials [35]. Although starch offers a sustainable alternative to petroleum-derived polymers, its films often exhibit limited mechanical strength and distinct rigidity [36,37], prompting research into polymer additives to enhance performance [38]. In the synthetic polymer industry, polyvinyl alcohol (PVA) is extensively produced and valued for its biodegradability, chemical resistance, and remarkable mechanical properties [36]. This material has demonstrated excellent film-forming ability, substantial stability at high temperatures, and strong adhesion [39]. However, PVA suffers from poor dimensional stability due to its water absorption capacity [40]. Given the above, a practical strategy involves incorporating PVA into natural polymer formulations, resulting in improved mechanical properties and reduced production costs in the sector [41].

Despite the promising mechanical and biodegradable properties of casein–starch films, their poor water vapor barrier performance continues to limit their broader application in food packaging [10]. In response, recent studies have focused on nanocomposites that offer enhanced resistance to water vapor and gas transmission, including N_2_, O_2_, and CO_2_ molecules [42]. Among these, nanoclays have emerged as abundant nanoscale additives capable of reinforcing polymer matrices by improving mechanical strength, thermal stability, and barrier properties [43]. One of the most promising candidates is bentonite, a clay mineral formed from volcanic ash, known for its swelling capacity, water retention, and layered silicate structure [44]. The hydrophilic nature of clay minerals enables the absorption of water molecules between interlayer spaces, where exchangeable cations reside, contributing to improved film performance even at low loading levels (<5 wt%) [42,45]. The integration of clay into polymer systems has been successfully demonstrated in both starch-based [46,47,48] and casein-based films [49,50]. However, multicomponent systems to better understand the effects of bentonite clay nanofillers on various functional properties of packaging films remain underexplored. This study examines the role of bentonite in enhancing the functional properties—mechanical, optical, barrier, and microbiological—of a biodegradable composite film comprising calcium caseinate, starch, and PVA. Through this integrated approach, this research contributes to the advancement of multifunctional packaging systems with improved sustainability and performance.

## 2. Materials and Methods

### 2.1. Materials

Spray-dried calcium caseinate (CAS) powder containing 92.1% protein was obtained from Centro Agrolechero Group S.A.S. (Cra. 14a #71-35, Bogota, Colombia). Additional materials included snowflake-modified starch (STA), PVA with a molecular weight of 89,000–98,000 MW and a hydrolysis degree above 99%, and bentonite clay (BENT) with a density of 4.23 g/cm^3^, all purchased from Químicos Campota y Cía Ltd.a (Calle 13 # 13-27, Bogota, Colombia).

### 2.2. Preparation of CAS and STA Dispersions

A 6% (*w*/*v*) CAS solution in distilled water (DW) was prepared at an ambient temperature and heated to 35 °C, followed by the addition of 1.0 M NaOH solution (equivalent to 30% *v*/*v* of the caseinate solution) to neutralize the dispersion, as described in previous methodologies [30,51]. Stirring was maintained using a magnetic stirrer, followed by the addition of glycerol (GLY) at 30% (*w*/*w*) relative to CAS content.

Separately, a 5% (*w*/*v*) STA solution was prepared in DW and subsequently heated to 90 °C. GLY was then added at 40% (*w*/*w*), corresponding to the STA content. The mixture was stirred at 500 rpm for 20 min until complete gelatinization was achieved.

### 2.3. Preparation of BENT Suspension

A 4% (*w*/*v*) BENT solution was prepared by dispersing the clay in DW under continuous magnetic stirring. To ensure uniform dispersion, the mixture was subjected to ultrasonic treatment at 40 kHz for 1 h at room temperature. Following sonication, BENT suspension was incorporated into the polymer solution.

### 2.4. Design of Biodegradable Film System

The CAS solution was combined with the STA solution at a defined volumetric ratio and manually stirred to achieve homogeneity. Base formulations were prepared to evaluate the effect of different casein/starch ratios on film performance (Table 1). These combinations were selected based on preliminary screening tests aimed at identifying an optimal formulation with improved mechanical strength. Each treatment was prepared in triplicate, and tensile strength measurements were obtained from at least eight individual film samples per formulation (*n* ≥ 8). Among the tested formulations, the sample containing a 2:1 ratio of casein to starch (1.8 g casein/0.9 g starch) consistently exhibited the highest tensile strength across replicates. This formulation was therefore selected as the optimal base for the study.

The final step involved the inclusion of a 6% (*w*/*v*) PVA solution prepared in DW, which was stirred continuously for 1 h at 80 °C. This solution was then left to rest at ambient conditions for 24 h. To complete the final polymer formulation, the CAS/STA/BENT mixture was combined with the PVA solution at a volumetric ratio of 30:70, respectively. The resulting composite solution (CAS/STA/GLY/PVA/BENT) was gently heated to 35 °C and subjected to vacuum degassing to eliminate entrapped air bubbles. For film casting, 15 mL of the degassed solution was poured into polystyrene Petri dishes and allowed to dry in an oven at 38 °C for 24 h. Once dried, the films were stored in a desiccator for 48 h to stabilize the moisture content.

### 2.5. Determination of Final Polymer Viscosity

Viscosity measurements of the final film-forming solution were conducted using a Brookfield DV-II+ Pro programmable viscometer. This parameter is crucial for evaluating the energy requirements associated with potential extrusion-based optimization of the film. The analysis was carried out at 25 °C, with the spindle speed progressively increased during testing. Only readings corresponding to torque values exceeding 10% were considered.

### 2.6. Bio-Nanocomposite Film Characterization

#### 2.6.1. Fourier-Transform Infrared (FTIR) Spectroscopy

FTIR analysis was conducted to investigate molecular interactions and confirm the compatibility among the polymeric components. Using an FTIR spectrometer (Agilent Cary 630), spectral data for each sample were acquired across a wavenumber range of 400–4000 cm^−1^.

#### 2.6.2. Surface Microstructure

The BENT morphology and the nanostructured biodegradable films were examined using a high-resolution field emission scanning electron microscope (FE-SEM) (TESCAN LYRA3) with a focused gallium ion beam source. SEM was conducted at 15 kV, and all samples were sectioned, coated, and mounted on aluminum stubs. Additionally, the chemical composition was evaluated using energy-dispersive X-ray spectroscopy (EDS).

#### 2.6.3. Mechanical Properties

Film thickness values were obtained using a digital DC1004-6 caliper at five randomly selected points per sample, with the average estimated for each group of samples (n = 8). Mechanical performance was evaluated in accordance with the ASTM D882 standard using a 0.2 kN load WDW-30 universal testing machine (Jinan Testing Equipment IE Corporation, China). Samples were cut (2 cm × 8 cm) and tested at 50 mm/min, reporting data for each sample (n = 8).

#### 2.6.4. Color Analysis

The color characterization of the films was evaluated using a CR-410 Chroma Meter instrument. Each sample was positioned within the measurement head, and the color parameters L* (lightness), a* (red–green axis), b* (yellow–blue axis), chroma (C*), and hue angle (h°) were recorded across four replicates. The final formulation film (CAS/STA/GLY/PVA/BENT) and one without BENT were analyzed for comparative assessment. Film specimens were cut into rectangular shapes and placed inside the sample holder of a Thermo Scientific Evolution 300 UV–Vis spectrophotometer, equipped with a xenon lamp. For this light barrier characterization, absorbance spectra were recorded over the wavelength range of 400–800 nm. As described in similar studies [52,53], transparency was evaluated at 600 nm in duplicate using Equation (1):

(1) $$Transparency=\frac{\log(\%T_{600})}{x}$$

According to the ASTM D1003-00 standard and previous procedures [54,55], the optical opacity of the films was expressed as absorbance multiplied by wavelength (AU·nm). The value was obtained through the determination of the area under the recorded curve, which quantifies the total absorbance across the measured wavelength range.

#### 2.6.5. Water Vapor Permeability (WVP) Determination

WVP was assessed in a well-sealed system using a gravimetric test following ASTM E96 [56]. Samples were attached over borosilicate glass beakers containing DW and secured to their openings using rubber bands. These setups were placed inside a desiccator containing silica gel to maintain a dry environment. The system was maintained at 21 °C and 60% relative humidity. The samples were weighed at 12 h intervals over a three-day period to monitor moisture loss. The WVP values were determined using the following expression:

(2) $$WVP=\frac{\left(\frac{g}{t}\right)*i}{A*∆P}$$

where *g/t* is the rate of weight loss, determined from the slope of a simple linear regression; i is the thickness measurement (m); *A* is the film surface area (m^2^); and ∆*P* is the water vapor partial pressure difference across the film (Pa).

#### 2.6.6. Microbiological Assessment

Two microbiological assays were conducted for the evaluation of the potential effect of the developed films as packaging materials. Following the ASTM D4635-86 standard, film samples were placed over Petri dishes containing Tryptic Soy Agar (TSA) and tested in triplicate. Every dish was taken to incubation (30 °C) for 24–48 h, after which the total number of bacterial colonies was recorded. For fungal analysis, samples were placed directly over Potato Dextrose Agar (PDA) plates and incubated at 25 °C for 5 days. Colony growth on the film surface was monitored to assess microbial inhibition.

#### 2.6.7. Biodegradation Rate

A biodegradability test was proposed using a soil burial method adapted from a previously reported procedure [57]. Rectangular samples were cut (2 × 3 cm^2^), and their initial weight (*W*_0_) was determined after a 2 h drying process at 90 °C. Each sample was then placed in an aluminum cup and buried at a depth of 5 cm in 6.122 kg of indoor soil contained within a container (30 × 11 × 13 cm^3^). The experiment was conducted at 21 °C over a 9-day period. Samples were retrieved every 24 h, gently cleaned to remove soil residues, and dried at 80 °C for 2 h before weighing. The average weight loss (*W_t_*) was used to calculate the biodegradation rate according to Equation (3):

(3) $$\%WL=\left[\frac{W_{o}-W_{t}}{W_{o}}\right]\times \;100$$

## 3. Results and Discussion

### 3.1. Viscosity of the Final Solution

**Figure 1:**
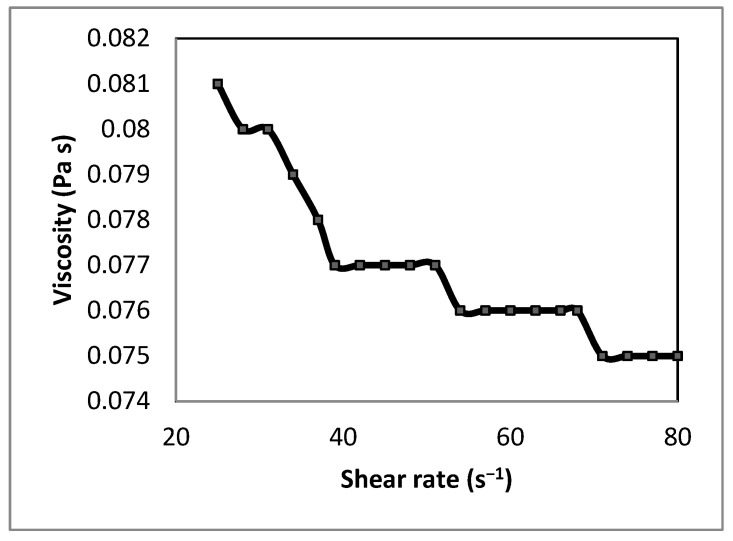
Viscosity vs. shear rate of polymeric solution of CAS/STA/GLY/PVA/BENT.

The viscosity of the final polymeric solution was evaluated to assess its flow behavior. Figure 1 illustrates a decrease in viscosity from approximately 0.081 Pa·s to 0.075 Pa·s as the shear rate increased from 30 to 75 s^−1^, indicative of pseudoplastic (shear-thinning) behavior in the CAS/STA/GLY/PVA/BENT solution. This trend is attributed to an increase in molecular kinetic energy [58] and the alignment of polymer chains [59]. Therefore, there is a reduction in resistance as entangled molecules begin to detangle and orient in the direction of flow [60,61]. The average viscosity, measured at ~0.077 Pa·s, exceeds values typically observed for pure PVA solutions at comparable concentrations (5–10 wt%), which range from ~0.005 to 0.015 Pa·s [62]. This increase is likely due to the presence of casein and starch, which promote molecular entanglement and form compatible interactions via hydrogen bonding [63].

Although glycerol was incorporated during the solubilization of casein and starch, its presence in the final film-forming solution remained relatively low compared to the PVA proportion. Prior research indicates that plasticizers such as glycerol can lower the viscosity of PVA-based systems by promoting polymer chain mobility [64]. Since PVA solutions are dissolved in water, water molecules can penetrate the PVA chains more easily, thereby improving the flow behavior [65]. In the present formulation, the dominant PVA and the biopolymer network likely restricted the plasticizing effect of glycerol, resulting in higher viscosity and a more entangled matrix. The selected PVA content ensured a consistent film by maintaining stable clay dispersion, while clay itself has been shown to enhance rheological control for efficient casting [66]. The good processability of the nanocomposite solution supports film-forming applications and other uses where these viscous polymer solutions can be utilized, such as electrospinning techniques and nanofiber production [67].

### 3.2. FTIR Spectra

**Figure 2:**
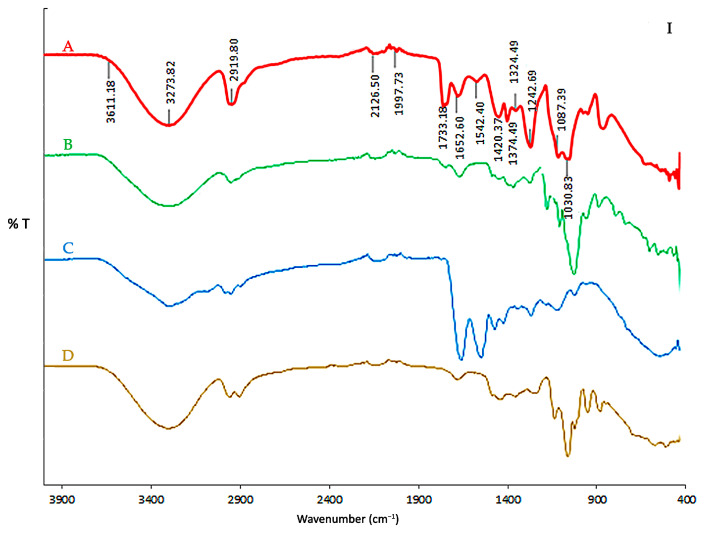

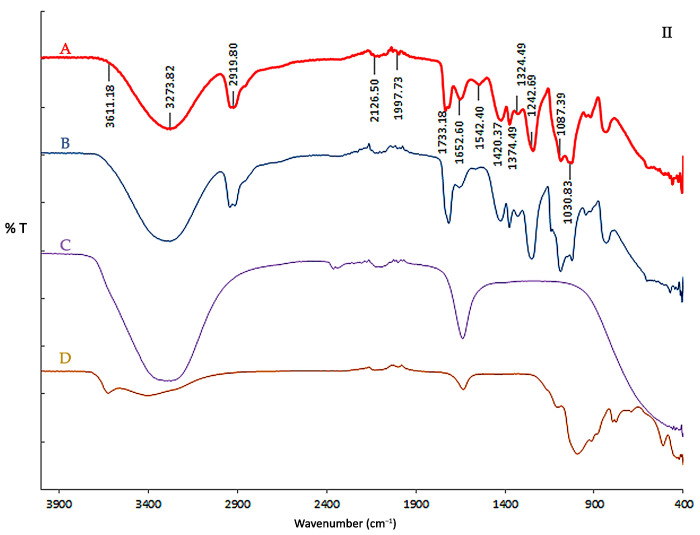
FTIR spectra of individual components and the final biodegradable film. (**I**) Spectra of (**A**) final film (CAS/STA/GLY/PVA/BENT), (**B**) modified starch (STA), (**C**) calcium caseinate (CAS), and (**D**) glycerol (GLY). (**II**) Spectra of (**A**) final film, (**B**) polyvinyl alcohol (PVA), (**C**) sodium hydroxide (NaOH), and (**D**) bentonite (BENT).

Figure 2 shows the FTIR spectra of the biodegradable final film and its components. The broad O–H stretching band (~3273 cm^−1^) observed in all spectra is attributed to strong hydrogen bonding [68] among casein, starch, PVA, glycerol, and bentonite. The film’s characteristic peaks at 2920–2850 cm^−1^ correspond to C–H stretching (from proteins and PVA), while the C=O stretching band at 1733 cm^−1^ indicates carbonyl groups arising from protein and plasticizer interactions [69].

Protein-related amide bands [29,70] are observed at 1634 cm^−1^ and 1515 cm^−1^ (from casein) and are retained in the final film. The glycerol and starch C–O stretching bands (~1030–996 cm^−1^) are also visible, with minimal shifts, suggesting physical rather than chemical interactions [71,72]. The FTIR spectrum of bentonite is shown in Figure 2II with characteristic peaks at 3623 cm^−1^ (O–H stretch) and 1089 cm^−1^ corresponding to Si–O symmetric stretching [73]. Finally, a slight shift in the final film to 3611 cm^−1^ and 1087 cm^−1^ indicates hydrogen bonds between the clay and components of the polymer matrix [74], supporting the formation of a nanocomposite.

### 3.3. Surface Microstructure

**Figure 3:**
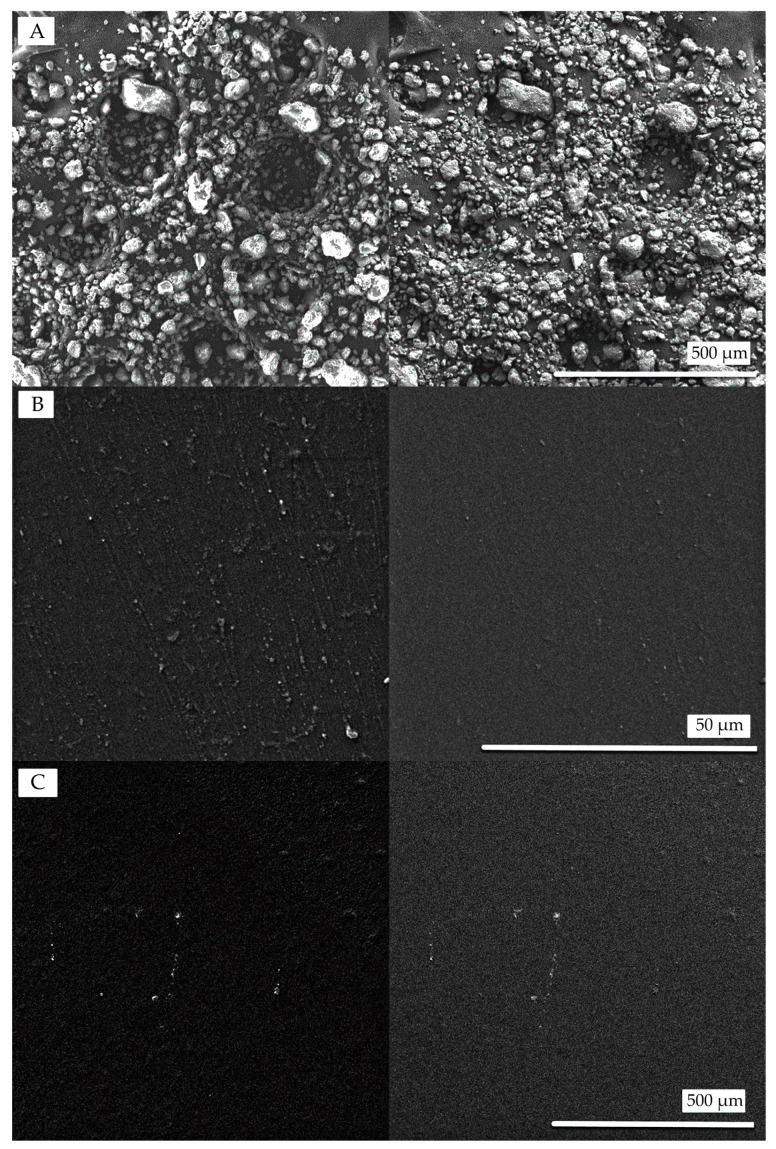
Scanning electron microscopy (SEM) micrographs of (**A**) pure bentonite, (**B**) the CAS/STA/GLY/PVA film without clay, and (**C**) the nanocomposite CAS/STA/GLY/PVA/BENT film. BSE (left) and SE (right) images highlight compositional and surface morphology differences across samples.

An SEM analysis was performed to provide qualitative insight into surface morphology and to examine any visible effects of bentonite integration within the composite films. Figure 3 illustrates the morphological features of bentonite and various film samples as observed through SEM using both secondary electron (SE) and backscattered electron (BSE) imaging modes. Figure 3A presents the native surface structure of bentonite prior to incorporation. In the clay-free film shown in Figure 3B, the surface appears relatively smooth with few irregularities, though the dispersed particulate regions may indicate partial miscibility of casein within the starch matrix [75]. In contrast, the sample in Figure 3C with bentonite exhibits a rougher texture, suggesting dispersion throughout the matrix driven by PVA–clay affinity [76]. Visible surface irregularities, attributed to agglomerated clay particles [77], indicate partial exfoliation rather than complete dispersion, confirming the incorporation of silicate structures [78]. This increase in surface roughness aligns with similar findings comparing film morphology across different compositions [79]. While high-resolution imaging was beyond the scope of this study, the SEM imaging indicates nanoclay presence and interaction; future work could employ transmission electron microscopy (TEM) or cross-sectional SEM to better characterize dispersion within the matrix.

**Figure 4:**
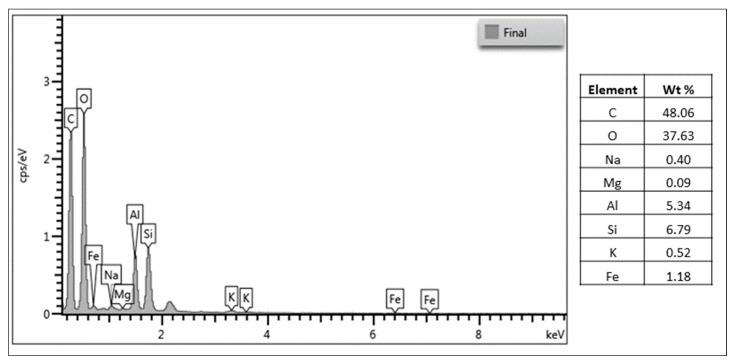
EDS spectrum and elemental composition of the nanostructured CAS/STA/GLY/PVA/BENT film.

Furthermore, Figure 4 presents the EDS analysis of the nanostructured CAS/STA/GLY/PVA/BENT film, revealing its elemental composition. Dominant peaks corresponding to carbon (48.06 wt%) and oxygen (37.63 wt%) are consistent with the organic biopolymer matrix composed of casein, starch, glycerol, and PVA. Notably, the presence of silicon (6.79 wt%), aluminum (5.34 wt%), and iron (1.18 wt%) confirms the successful incorporation of bentonite, despite its low content (4 wt%). These elements are characteristic of clay minerals, which are primarily composed of alumina, silica, and various oxides [80,81]. The detection of such peaks at low concentrations suggests that bentonite was dispersed throughout the polymer network, demonstrating the effective integration of clay into the film’s composition [82].

### 3.4. Mechanical Properties

Table 2 presents a comparative summary of mechanical properties, including thickness, tensile strength, elongation at break, and Young’s modulus for the CAS/STA/GLY/PVA/BENT film developed in this study, alongside analogous biopolymer-based films reported in the literature. The reference films were selected based on compositional similarity, particularly the inclusion of biopolymers such as casein (CAS) and starch (STA), along with common additives including glycerol (GLY), polyvinyl alcohol (PVA), and clay-based materials, or functionally related proteins such as whey protein isolate (WPI). The final formulation demonstrated strong mechanical performance, with a tensile strength of 13.08 ± 2.11 MPa and an elongation at break of 109.30 ± 0.08%, outperforming most reference materials in both strength and flexibility. In this comparison, the CAS/GLY/PVA film [10] displayed a higher elongation (275%) due to the absence of clay. In contrast, one of the most comparable CAS/STA/GLY/CLAY [50] formulations reached 9.92 ± 1.27 MPa with 92.33 ± 19.21% elongation—both lower than the values obtained in this study.

The enhancement in tensile strength (13.08 ± 2.11 MPa) is attributed to the addition of bentonite. Clay-reinforced films, even with low filler concentrations (<0.05 wt%), have demonstrated notable improvements in mechanical performance due to better stress transfer within the polymer matrix [84]. The significant Young’s modulus observed is mainly due to the montmorillonite component of bentonite, whose extensive surface area and layered silicate structure impede polymer chain movement and enhance matrix rigidity [85,86].

Plasticizer content also influences mechanical behavior. Films containing glycerol typically showed reduced tensile strength due to the disruption of intermolecular forces, as seen in CAS/GLY/WPC [84] with a low strength of 3.40 ± 0.59 MPa and moderate elongation (55.30 ± 1.83%). In contrast, the incorporation of PVA improves compatibility with other polymers [53,87], contributing to an approximate 32% increase in tensile strength relative to the STA/GLY/PVA/CLAY film [83], which achieved only 12.41 ± 4.19 MPa and exhibited limited elongation (3.20 ± 0.81%). These results clearly demonstrate that the inclusion of bentonite and PVA reinforces the film’s mechanical profile, achieving a combination of rigidity and stretchability—a balanced performance for practical use in food packaging.

### 3.5. Optical Characterization

Color plays a pivotal role in influencing consumer perception and defining visual elements across all forms of food packaging [88]. As shown in Table 3, the inclusion of bentonite induced visible alterations evident in the color parameters analyzed. The CAS/STA/GLY/PVA/BENT sample exhibited a higher chroma (C* = 2.8 ± 0.08) compared to the clay-free counterpart (C = 1.21 ± 1.77), indicating greater color saturation associated with bentonite’s presence. The shift in b* values (−1.12 vs. −2.76) reflects a reduction in blue tones, consistent with the PVA’s color contribution and the clay’s natural yellowish appearance. This transition toward yellow is attributed to Cloisite Na+ pigmentation [89], and other studies have reported nanoclay integration increasing yellow coloration in films [50,90]. Meanwhile, the a* values remained positive, indicating a minimal red tint across both films.

Additionally, the hue angle (h°) slightly decreased from 282.93° ± 2.95 (CAS/STA/GLY/PVA) to 278.65° ± 3.77 (CAS/STA/GLY/PVA/BENT), reinforcing a minor shift in color direction likely due to bentonite’s influence. A slight reduction in lightness (L*) and an increase in chroma further demonstrate the clay’s effect on visual appearance, with these findings aligning with previously reported profiles of biodegradable films containing nanoclays [91].

**Figure 5:**
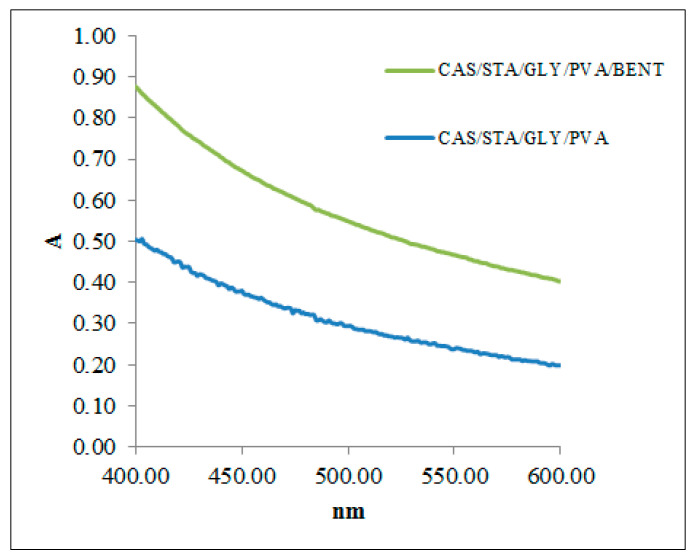
UV–Vis absorbance spectra of biopolymer films with (CAS/STA/GLY/PVA/BENT) and without bentonite (CAS/STA/GLY/PVA) across the 400–600 nm range.

The light barrier performance of the developed films is presented in Table 4. Opacity is an established indicator of transparency and plays a role in protecting packaged food from light-related quality degradation [92]. As illustrated in Figure 5, the UV–Vis absorbance spectra revealed that the clay-containing film (CAS/STA/GLY/PVA/BENT) steadily exhibited higher absorbance values in the 400–600 nm range compared to the bentonite-free sample, confirming its superior light barrier performance. This effect is attributed to bentonite’s layered silicate structure, which increases light scattering and reduces transmission [76].

Supporting this trend, Table 4 reports that the bentonite-based film demonstrated lower transmittance (44.37%) and higher opacity (181 ± 2 AU nm) than the film without clay (60.21%, 134.48 ± 0.53 AU nm). The addition of nanoclays, such as bentonite, is known to decrease UV transmittance—an enhancement that helps prevent UV-induced lipid oxidation and prolongs product shelf life [93]. Thus, films containing bentonite show promise for packaging light-sensitive fresh foods [94], offering strong UV protection while remaining transparent in the visible region.

### 3.6. Water Vapor Permeability (WVP) Assay

**Figure 6:**
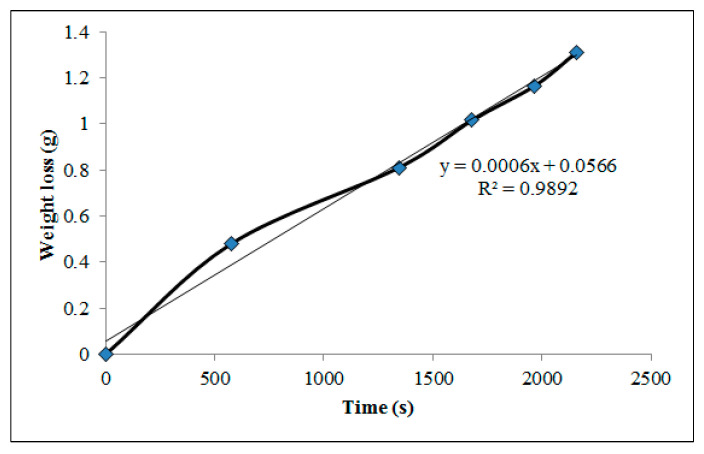
Weight loss over time used to determine the water vapor transmission rate of the CAS/STA/GLY/PVA/BENT film.

WVP is a critical parameter for edible and biodegradable packaging films, as it directly influences product shelf life and moisture protection [19]. Figure 6 illustrates the recorded weight loss over time, with a total reduction of 4.7%. PVA-based systems are typically associated with high WVP due to the polymer’s hydrophilic nature, allowing water molecules to penetrate easily through its matrix [10]. However, as shown in Table 5, the addition of bentonite significantly reduced WVP by approximately three orders of magnitude compared to conventional CAS/STA/GLY films [75]. This improvement is partially attributed to the limited use of plasticizer, as higher GLY concentrations can increase WVP in hygroscopic films by weakening intermolecular interactions [30].

The incorporation of bentonite contributes further to barrier performance, particularly due to the distribution of silicate layers with large aspect ratios [95]. These layers act as impermeable barriers, forcing water vapor to follow tortuous and extended diffusion pathways across the film matrix [91]. This structural arrangement minimizes moisture transfer and reinforces the overall barrier properties [54,83]. Similar WVP reduction rates have been reported for various types of these nanocomposites [96,97,98], representing the obstructive nature of dispersed clay layers. Altogether, these findings confirm that the presence of bentonite promotes the formation of a more compact and ordered nanocomposite network, making the film suitable for moisture-sensitive food packaging applications.

### 3.7. Microbiology Test

**Figure 7:**
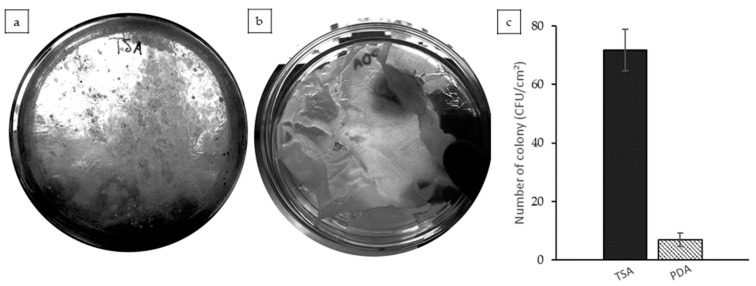
Microbiological evaluation of CAS/STA/GLY/PVA/BENT film after 48 h: (**a**) total aerobic bacterial count on TSA medium. (**b**) Mold and yeast growth on PDA medium with visible localized contamination. (**c**) Quantification of colony-forming units (CFU/cm^2^) based on plate counts.

Microbial evaluation of the CAS/STA/GLY/PVA/BENT films revealed 71.67 ± 7.1 CFU/cm^2^ of total aerobic bacteria (TSA) after incubation (Figure 7a). These values are within the general microbial load range reported for non-antimicrobial biodegradable packaging films [16]. While the films did not exhibit antimicrobial action, the microbial load remained below common hygiene standards. According to ISO 4833-1:2013 (for aerobic bacterial count) [99] and ISO 21527-1:2008 (for molds and yeasts) [100], counts below 10^2^ CFU/cm^2^ are typically acceptable in packaging applications not intended for sterile use. For the fungal growth experiment, 7.0 ± 2.3 CFU/cm^2^ was identified, and visible mold formation was likely due to airborne contamination. These findings highlight the importance of adhering to aseptic techniques during film preparation and testing. To enhance antimicrobial performance, future work should consider incorporating active agents into the film matrix. Notably, the inclusion of nanoclay in biopolymer systems has been shown to improve antimicrobial efficacy [101]; however, a more detailed and extensive microbiological assessment would be required to verify the veracity of these antibacterial properties within the present system. Additionally, antibacterial mechanisms, such as the use of silver nanoparticles (AgNPs), have been employed to inhibit the growth of common pathogens in the food industry, demonstrating that their incorporation into nanocomposite films significantly enhances antibacterial activity [102,103]. Taken together, these insights suggest that the antimicrobial potential of the current film system could be validated and optimized through targeted incorporation of functional nanomaterials. Therefore, specific assays against foodborne pathogens should be pursued to confirm efficacy and guide future formulation strategies.

### 3.8. Biodegradation Rate

**Figure 8:**
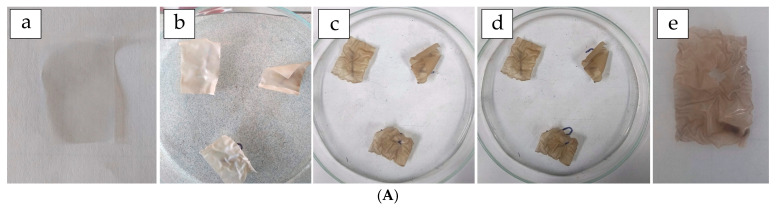

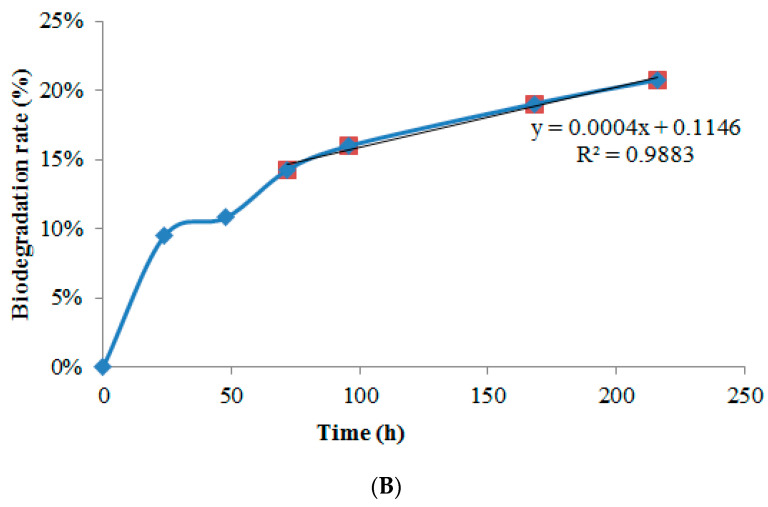
(**A**) Visual progression of film biodegradation at selected time points: (**a**) 0 h (0 weeks), (**b**) 24 h, (**c**) 168 h (7 days), (**d**) 192 h, and (**e**) 216 h (9 days). (**B**) Biodegradation profile of the CAS/STA/GLY/PVA/BENT film monitored during 10 days of burial in soil.

The biodegradability of the nanostructured film was monitored over a 9-day period through periodic weight measurements. In addition to progressive weight loss, visible physical deterioration and an increase in opacity were observed in the samples (Figure 8). The most rapid degradation occurred within the first 72 h, after which the rate followed a more linear trend. Previous studies have reported lower biodegradation rates (approximately 20% over three weeks) for films containing PVA and starch [48]. In contrast, the inclusion of biopolymers in the present film formulation appears to support improved biodegradability, particularly when the film is subjected to soil burial.

As shown in Figure 8B, the CAS/STA/GLY/PVA/BENT film exhibited a steady increase in weight loss. Based on the extrapolation of the degradation curve, complete biodegradation is estimated to occur around 13 weeks (≈2213.5 h). Although this timeline exceeds those reported for simpler casein–starch systems, the findings confirm the biodegradability of the composite, even with the inclusion of poly (vinyl alcohol) (PVA) and bentonite. The slightly delayed degradation may be attributed to increased matrix cohesion resulting from protein–PVA interactions and partial crosslinking, which can reduce water diffusion and microbial access to the polymer chains [50,104]. Moreover, nanoclays like bentonite—while beneficial for mechanical and barrier properties—can reduce biodegradation rates by impeding structural breakdown [83,105]. Overall, these experiments confirm the film’s potential for controlled biodegradation, a characteristic that aligns well with the sustainability goals of modern food packaging. This eventual environmental breakdown of such materials contributes significantly to the design of innovative biopolymer systems.

## 4. Conclusions

A biodegradable film composed of calcium caseinate and modified starch (2:1 ratio) was developed and enhanced with a nanoclay compound. The incorporation of bentonite significantly improved the film’s mechanical properties, achieving a tensile strength of 13.08 MPa and an elongation at break of 109.3%, outperforming similar films without polyvinyl alcohol (PVA). FTIR analysis confirmed the presence of physical and hydrogen bonding interactions among the film components, supporting the formation of a stable nanocomposite matrix. SEM imaging revealed a textured film surface, roughness attributed to the inclusion of bentonite nanoclay. Although optical transparency decreased slightly, the change in opacity remained imperceptible to the naked eye, suggesting no adverse impact on visual presentation. Water vapor permeability was reduced by approximately two orders of magnitude compared to similar formulations reported in the literature, highlighting the effectiveness of the nanostructured matrix in enhancing the moisture barrier properties. Microbiological assays showed bacterial levels within acceptable limits for casein-based films used in food packaging. However, further antimicrobial evaluation is recommended, and complementary thermal analysis should be conducted to confirm the material’s stability under varied temperature conditions. Lastly, the biodegradability assessment demonstrated that the film is capable of complete disintegration in soil within approximately 13 weeks, based on extrapolation of the degradation curve, thereby supporting its potential as a sustainable substitute for conventional plastic packaging.

## Figures and Tables

**Table 1:** Composition of the tested casein/starch formulations.

Casein [g]	Starch [g]	Casein/Starch Ratio
0.9	0	1:0
1.8	0.9	2:1
0.9	0.9	1:1
0.9	1.8	1:2
0	0.9	0:1

**Table 2:** Mechanical properties of CAS/STA/GLY/PVA/BENT nanocomposite film compared to similar biopolymer films from the literature.

Sample	Thickness (mm)	Tensile Strength, (MPa)	Tensile Strain, %	Young’s Modulus (MPa)	Reference
CAS/STA/GLY/PVA/BENT *	0.09 ± 0.01	13.08 ± 2.11	109.30 ± 0.08	11.73 ± 0.96	This work
CAS/GLY/PVA	-	19	275	-	[10]
CAS/STA/GLY/CLAY	0.13 ± 0.003	9.92 ± 1.27	92.33 ± 19.21	-	[50]
STA/GLY/PVA/CLAY	-	12.41 ± 4.19	3.20 ± 0.81	-	[83]
CAS/GLY/WPI	0.19 ± 0.05	3.40 ± 0.59	55.30 ± 1.83	5.50 ± 1.13	[84]

* Mean ± standard deviation (n = 8). - Value not reported in the original source. WPI: whey protein isolate (WPI).

**Table 3:** Color parameters of CAS/STA/GLY/PVA films with and without bentonite: lightness (L*), red–green coordinate (a*), blue–yellow coordinate (b*), chroma (C*), and hue angle (h°).

Sample	L*	a*	b*	C*	h°
CAS/STA/GLY/PVA	39.74 ± 0.44	0.40 ± 0.10	−2.76 ± 0.09	1.21 ± 1.77	282.932 ± 2.95
CAS/STA/GLY/PVA/BENT	38.94 ± 0.77	0.42 ± 0.18	−1.12 ± 1.55	2.8 ± 0.08	278.65 ± 3.77

Values are expressed as mean ± standard deviation (*n* = 4).

**Table 4:** Light barrier properties and opacity of CAS/STA/GLY/PVA films with and without bentonite.

Sample	Absorbance	%T	Thickness (mm)	Transparency	Opacity (AU nm)
CAS/STA/GLY/PVA	0.25	60.206	0.1016	17.52 ± 0.77	134.48 ± 0.527
CAS/STA/GLY/PVA/BENT	0.36	44.370	0.1016	16.21 ± 0.434	181 ± 2.0

Values are expressed as mean ± standard deviation (*n* = 2).

**Table 5:** Water vapor permeability (WVP) parameters of CAS/STA/GLY/PVA/BENT film.

SLOPE (g/s)	i (m)	A (m^2^)	∆P (Pa)	WVP (g/m·s·Pa)
6.00 × 10^−4^	1.02 × 10^−4^	3.14 × 10^−4^	2.49 × 10^3^	8.23 × 10^−8^

Note: film thickness (i), exposed area of film (A), partial pressure difference (∆P), and (g/t) slope from weight loss over time.

## Data Availability

The original contributions presented in this study are included in the article. Further inquiries can be directed to the corresponding authors.
